# ALV-J GP37 Molecular Analysis Reveals Novel Virus-Adapted Sites and Three Tyrosine-Based Env Species

**DOI:** 10.1371/journal.pone.0122887

**Published:** 2015-04-07

**Authors:** Jianqiang Ye, Zhonglei Fan, Jianjun Shang, Xiaoyan Tian, Jialiang Yang, Hongjun Chen, Hongxia Shao, Aijian Qin

**Affiliations:** 1 Ministry of Education Key Laboratory for Avian Preventive Medicine, Yangzhou University, Yangzhou, Jiangsu, P. R. China; 2 Key Laboratory of Jiangsu Preventive Veterinary Medicine, Yangzhou University, Yangzhou, Jiangsu, P. R. China; 3 Jiangsu Co-innovation Center for Prevention and Control of Important Animal Infectious Diseases and Zoonoses, Yangzhou, Jiangsu, P. R. China; 4 Institute of Genomics and Multiscale Biology, Icahn School of Medicine at Mount Sinai, NY, United States of America; 5 Shanghai Veterinary Research Institute, Chinese Academy of Agricultural Sciences, Shanghai, P. R. China; University of Texas at San Antonio, UNITED STATES

## Abstract

Compared to other avian leukosis viruses (ALV), ALV-J primarily induces myeloid leukemia and hemangioma and causes significant economic loss for the poultry industry. The ALV-J Env protein is hypothesized to be related to its unique pathogenesis. However, the molecular determinants of Env for ALV-J pathogenesis are unclear. In this study, we compared and analyzed GP37 of ALV-J Env and the EAV-HP sequence, which has high homology to that of ALV-J Env. Phylogenetic analysis revealed five groups of ALV-J GP37 and two novel ALV-J Envs with endemic GP85 and EAV-HP-like GP37. Furthermore, at least 15 virus-adapted mutations were detected in GP37 compared to the EAV-HP sequence. Further analysis demonstrated that three tyrosine-based motifs (YxxM, ITIM (immune tyrosine-based inhibitory motif) and ITAM-like (immune tyrosine-based active motif like)) associated with immune disease and oncogenesis were found in the cytoplasmic tail of GP37. Based on the potential function and distribution of these motifs in GP37, ALV-J Env was grouped into three species, inhibitory Env, bifunctional Env and active Env. Accordingly, 36.91%, 61.74% and 1.34% of ALV-J Env sequences from GenBank are classified as inhibitory, bifunctional and active Env, respectively. Additionally, the Env of the ALV-J prototype strain, HPRS-103, and 17 of 18 EAV-HP sequences belong to the inhibitory Env. And models for signal transduction of the three ALV-J Env species were predicted. Our findings and models provide novel insights for identifying the roles and molecular mechanism of ALV-J Env in the unique pathogenesis of ALV-J.

## Introduction

Avian leukosis virus (ALV) is an alpharetrovirus. Based on its antigenicity, interference and host range, chicken ALV is classified into six subgroups, including A, B, C, D, E and J [1, 2]. Unlike other subgroups, ALV-J infection induces hematopoietic malignancy with myeloid leukemia and hemangioma and causes significant economic loss for the poultry industry worldwide [3, 4]. However, little is known about the mechanism of the unique pathogenesis of ALV-J. Although the envelope protein (Env) plays vital roles in ALV-J pathogenesis [5, 6], the molecular determinants in Env for ALV-J pathogenesis require investigation. ALV-J Env only has approximately 40% homology with other ALVs, whereas it has approximately 97% identity to the endogenous EAV-HP sequence in the chicken genome [7]. Additionally, the genetic variation or evolution between EAV-HP and ALV-J Env during host adaptation remains unclear.

Similar to other retroviruses, the ALV-J Env protein contains two parts, GP85 and GP37 [1]. In ALV-J infected cells or in viral particles, GP85 is exposed to the cell or virus surface, whereas GP37 is anchored in the cell or viral membrane. Because GP85 plays vital roles in determining the viral host range and antigenicity, sequences of ALV-J GP85 from different isolates and species have been analyzed extensively in recent years [8–12]. However, the genetic diversity of ALV-J GP37 has not been systematically documented. In addition to mediating viral entry into cells, GP37 newly synthesized in the infected cells may provide potential docking sites for interaction with cellular proteins via its cytoplasmic domain (CTD). Therefore, similar to GP85, GP37 also plays critical roles in ALV-J infection, pathogenesis and host adaptation. In this study, we first summarize the isolation of ALV-J in our laboratory from 1999 to 2013 and then collect and analyze all GP37s of ALV-J Env and EAV-HP sequences deposited in GenBank. We report for the first time five groups of ALV-J GP37, 15 virus-adapted mutations, and the classification of ALV-J Env into three species based three tyrosine-based motifs in GP37.

## Materials and Methods

### Ethics Statement

**Fig 1:**
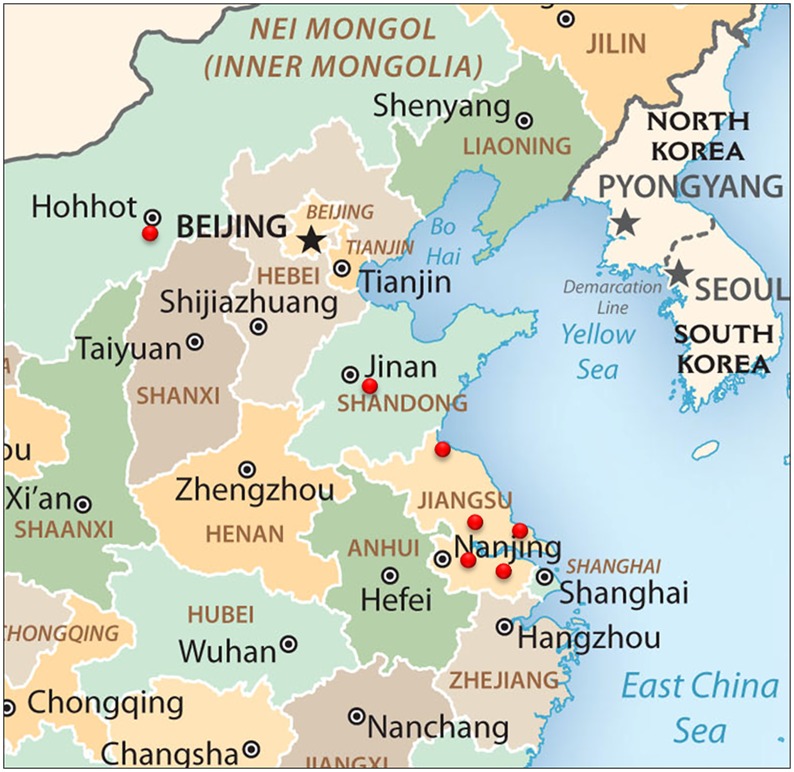
Location of these farms. The farm locations from where these samples collected for ALV-J isolation in this study were labeled as red dot in the map provided at the CIA.

All animal experiments were performed in accordance with the institutional animal care guidelines, and the protocol, #06R015, was approved by the Animal Care Committee at Yangzhou University in China. All farms are used for breeding meat-type or egg-type chickens and do not belong to any national park or other protected land or sea area. Fourteen, two and one chicken farms were from Jiangsu, Shangdong and Neimeng province in China, respectively, as described in Table 1 and the coordination for the locations of these farms were shown in Fig 1. And these studies did not involve endangered or protected species and were performed in the Key Laboratory for Avian Preventive Medicine of the Ministry of Education and the Key Laboratory of Jiangsu Preventive Veterinary Medicine at Yangzhou University in China.

**Table 1:** Summary of the ALV-J viruses isolated in our laboratory from 1999 to 2013.

No	Isolate	Sample from	Year	Chicken type	Accession no.
1	YZ9901	Jiangsu	1999	Meat type	AY897222
2	YZ9902	Jiangsu	1999	Meat type	HM235670
3	SD9901	Shangdong	1999	Meat type	AY897220
4	SD9902	Shangdong	1999	Meat type	AY897221
5	JS-nt	Jiangsu	2000	Meat type	HM235667
6	NM2002-1	Neimeng	2002	Meat type	HM235669
7	NHH	Jiangsu	2009	Egg type	HM235668
8	HAY013	Jiangsu	2009	Egg type	HM235665
9	JS09GY6	Jiangsu	2009	Egg type	GU982310
11	JS09GY5	Jiangsu	2009	Egg type	GU982309
12	JS09GY3	Jiangsu	2009	Egg type	GU982308
13	JS09GY2	Jiangsu	2009	Egg type	GU982307
14	JS13NJ01	Jiangsu	2013	Meat type	-
14	JS13NJ02	Jiangsu	2013	Meat type	-
15	JS13NJ03	Jiangsu	2013	Meat type	-
16	JS13YZ01	Jiangsu	2013	Meat type	-
17	JS13CZ01	Jiangsu	2013	Meat type	-

### Virus isolation and identification

Dead or sick chickens suspected of ALV-J infection from different farms during 1999–2013 were sent to our laboratory for diagnostics. In addition to the chickens, chicken blood samples were also sent to our laboratory for ALV-J diagnostics. Before we collected the organ samples, the sick chickens were killed by CO_2_ asphyxiation according to our protocol (#06R015), which was approved by the Animal Care Committee at Yangzhou University in China. For virus isolation, the samples, including kidney, liver, spleen and blood, were inoculated into DF1 cells. The inoculated cells with three blind passages were fixed and tested by immunofluorescence assay (IFA) with an ALV-J specific antibody JE-9, as previously described [13].

### PCR amplification

The ALV-J pro-viral genome was extracted for PCR detection as previously described [14]. The PCR amplicon covered the *env*, and the majority of the 3’LTR was sequenced by DNA sequencing. And the following primers were used for PCR: 5’-gctgccatcgagaggttact-3’ and 5’-agttgtcagggaatcgac-3’.

### Sequence analysis

In addition to the above ALV-J isolates, we collected all GP37s of ALV-J Env and EAV-HP deposited in GenBank. All sequences were aligned and analyzed using DNAStar. The phylogenetic tree was constructed using the neighbor-joining method (bootstrap1000 times) with MEGA6 [15]. The alignment of the selected positions in GP37 of ALV-J and EAV-HP were compared and generated by Weblogo [16].

## Results

### Summary of ALV-J virus isolation

A total of 17 ALV-Js were isolated in our laboratory using samples collected from different chicken farms during 1999–2013, as listed in Table 1. Eleven of the 17 isolates were isolated from meat-type chickens, and the others were from egg-type chickens. The isolation of the four isolates in 1999 was the first report of ALV-J in China [17]. Of these 17 isolates, five isolates were from blood samples obtained during our ALV route surveillance in 2013. The *env* gene of these isolates was sequenced and translated into protein. These ALV-J *env* genes from the earliest and the most recent Chinese ALV-J isolates provide useful sequence information for investigating the genetic diversity and evolution of ALV-J Env.

### Five groups of GP37 and two novel ALV-J Envs

**Fig 2:**
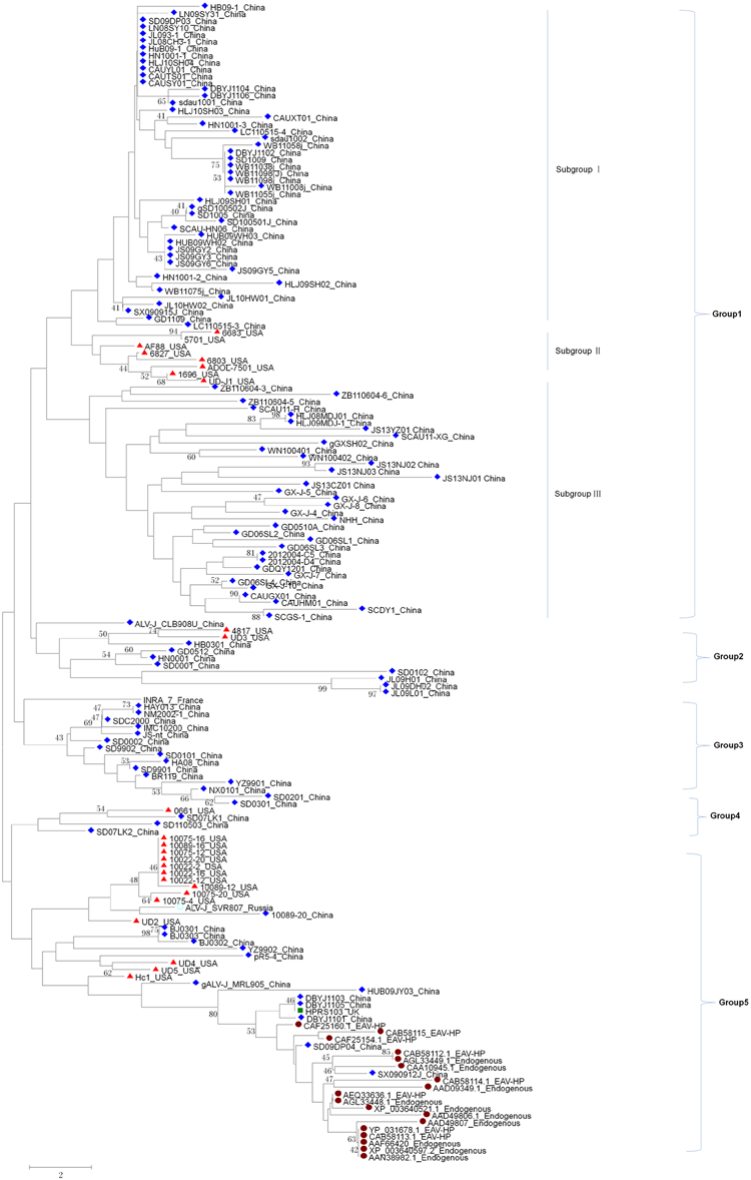
Phylogenetic tree of GP37 of ALV-J Env and EAV-HP. GP37 proteins (from site 1 to 193) of ALV-J Env and EAV-HP were aligned, and the GP37 phylogenetic tree was constructed using the neighbor-joining method (bootstrap 1000 times) with MEGA6. The label with red dot indicates the EAV-HP or other related endogenous sequence; the label with red angle indicates the USA ALV-J isolates; the label with blue rhombus indicates the Chinese ALV-J isolate; the label with green rectangle indicates the ALV-J prototype strain.

The phylogenetic tree analysis showed that all GP37s of ALV-J and EAV-HP tested were clustered into five groups, as described in Fig 2. Of these groups, Group 1 and Group 5 were the dominant groups. All EAV-HP GP37 sequences were clustered into Group 5. Group 5 also included the ALV-J prototype strain, HPRS-103, the first Chinese isolate, YZ9902, and the early USA isolates. In Group 5, although GP37 from two Chinese isolates SD09DP04 and SX090912J, ALV-J prototype HPRS-103, and EAV-HP were clustered together, the locations of the two Chinese isolates were within EAV-HP endogenous sequences whereas that of HPRS-103 presented outside EAV-HP endogenous sequences in the phylogenic tree. However, GP85 of the two ALV-J isolates was clustered closely with the endemic Chinese ALV-J isolates from egg-type chickens, such as SD09DP03 and SX090915J, and was far from the ALV-J prototype strain, as previously reported [8]. By contrast, GP37 of SD09DP03 and SX090915J were clustered into group 1, not in group 5 as described in Fig 2. Therefore, GP37 phylogenetic analysis reveals two novel ALV-J strains with endemic GP85 and EAV-HP-like GP37. In addition to EAV-HP and ALV-J GP37 in Group 5, all other ALV-J GP37 belonged to the other four groups, indicating the genetic diversity of ALV-J GP37 during viral evolution. Except for Group 3, the isolates from the USA and China were found in the other four groups. Group 1 could also be further clustered into three subgroups, namely, Subgroups I, II and III. Notably, ALV-J GP37 in Subgroup I and Subgroup III belonged to the Chinese isolates, whereas isolates in Subgroup II were from the USA. All these indicate that ALV-J GP37 in the Chinese isolates have more diversity than the USA isolates.

### Viral adapted sites in GP37 compared to EAV-HP

**Fig 3:**
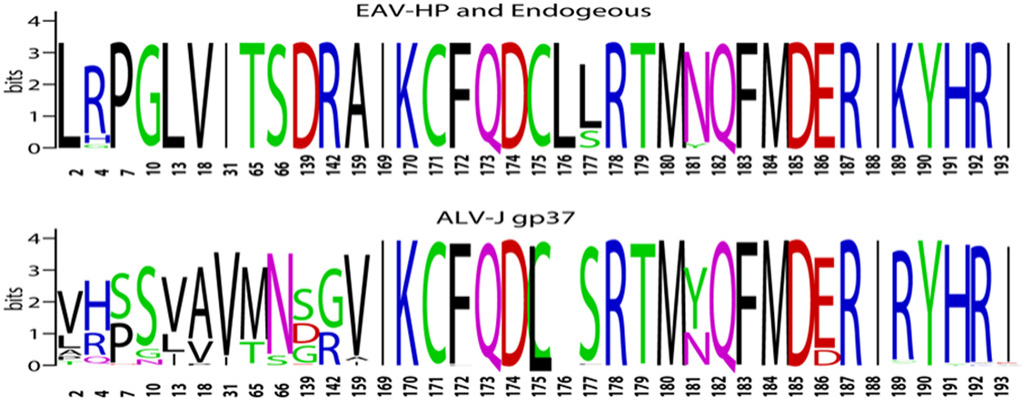
The alignment of the selected positions in GP37 of ALV-J and EAV-HP. The variation of the 15 virus-adapted sites and the cytoplasmic domain in GP37 of ALV-J and EAV- HP were compared and generated by Weblogo.

To test the variant sites and viral host adapted features, we compared the sites of ALV-J GP37 (from site 1 to 193) and the corresponding sequence of EAV-HP GP37. Our analysis showed that only 21 of 193 positions with variation were found in a total of 18 EAV-HP GP37 sequences, whereas 88 of 193 positions showed variation in a total of 149 ALV-J GP37 sequences. Compared to EAV-HP GP37, 15 of 193 positions in ALV-J GP37 showed variation in over 50% of ALV-J isolates, as described in Table 2 and Fig 3, indicating the viral host adaption of these sites. Notably, seven of these sites were located in a fusion peptide (sites 1–45), two were located in HR1 (sites 46–83), three were located in the transmembrane domain (sites 136–167), and three were located in the cytoplasmic domain (CTD) (sites 169–193). This suggests that these regions of GP37 play vital roles in viral host adaptation and evolution. In contrast to the other variant sites identified in Table 2 and Fig 3, position 189 with K was highly conserved in 18 EAV-HP GP37, but 189K was found in only one (isolate SD09DP04) of 149 ALV-J GP37 sequences. It should be noted that isolate SD09DP04 was also located in Group 5 and closely clustered within EAV-HP GP37. This indicates that 189K may be a novel site for differentiating EAV-HP GP37 from ALV-J GP37. Compared to the US isolates, several Chinese isolates also carried unique mutations, such as 57V and 186D. A total of four of five ALV-J viruses isolated from the blood samples in this study also carried 186D.

**Table 2:** Mutations in 15 sites of ALV-J GP37 compared to EAV-HP GP37.

Position	2	4	7	10	13
EAV-HP GP37	L (100%)	R (83%)	P (100%)	G (100%)	L (100%)
		H (11%)			
		G (6%)			
ALV-J GP37	L (20%)	R (26%)	P (46%)	G (11%)	L (23%)
	V (59%)	H (61%)	S (51%)	S (83%)	V (62%)
	A (9%)	Q (9%)	D (2%)	N (6%)	I (14%)
	P (6%)	L (1%)	A (1%)	Y (1%)	
	T (6%)	S (1%)			
	M (1%)	Y (1%)			
**Position**	**18**	**31**	**65**	**66**	**139**
EAV-HP GP37	V (100%)	I (100%)	T (100%)	S (100%)	D (100%)
ALV-J GP37	V (23%)	I (8%)	T (23%)	S (9%)	D (29%)
	A (74%)	V (92%)	M (74%)	N (91%)	S (43%)
	P (3%)		I (1%)		G (26%)
			L (1%)		E (2%)
			V (1%)		N (1%)
**Position**	**142**	**159**	**177**	**181**	**189**
EAV-HP GP37	R (100%)	A (100%)	L (78%)	N (94%)	K (100%)
			S (22%)	Y (6%)	
ALV-J GP37	R (35%)	A (4%)	L (1%)	N (36%)	K (1%)
	G (64%)	V (92%)	S (98%)	Y (63%)	R (95%)
	K (1%)	I (4%)	P (1%)	D (1%)	G (2%)
					S (1%)
					W (1%)

### Three tyrosine-based motifs present in GP37

We assessed the frequency of the 20 amino acids in these ALV-J and EAV-HP GP37 sequences and found that tyrosine was the scarcest residue of the 20 amino acids in these GP37 sequences. In EAV-HP GP37, only two positions (position 181 and 190) carried tyrosine. All 18 EAV-HP GP37 carried 190Y, whereas only one in 18 EAV-HPs carried 181Y and the other 17 EAV-HPs contained 181N. Interestingly, both positions 181 and 190 are located in the CTD of GP37. Moreover, 190Y was also highly conserved in all 149 ALV-J GP37s, and 181Y was found in 94 of 149 ALV-J GP37s. In addition to positions 181 and 190, tyrosine was also found in position 4 in one isolate, position 10 in one isolate, position 23 in one isolate and position 194 in two isolates from 149 ALV-J GP37 sequences. Because tyrosine located in the CTD of a membrane protein can be phosphorylated and plays vital roles in cellular signal transduction, the tyrosine in the CTD of ALV-J GP37 was further analyzed. Surprisingly, positions 181–184 in 94 of 149 ALV-J GP37s possessed a YQFM sequence as a YxxM motif, positions 188–193 in 147 of 149 ALV-J GP37s possessed an IRYHRI sequence as a S/I/V/LxYxxI/V/L motif, and position 181–197 in 2 of 149 ALV-J GP37s possessed an YQFMDERISYHAEYKKL sequence as a YxxI/Lx(6–12)YxxI/L-like motif. The X indicates any amino acid. Proteins containing phosphorylated YxxM can activate PI3K signaling, which is involved in cell growth, differentiation, survive and proliferation [18]. In the immune system, PI3K signaling is associated with immune deficiency, self-immunity and leukosis disease [19]. S/I/V/LxYxxI/V/L is the typical conserved sequence of the immune tyrosine-based inhibitory motif (ITIM), whereas YxxI/Lx(6–12)YxxI/L is the typical conserved sequence of the immune tyrosine-based active motif (ITAM) [20, 21]. The phosphorylated ITIM and ITAM can transduce inhibitory and activation signals to inhibit and stimulate immune cells, respectively [22, 23]. The alignment of the CTD (sites 169–193) of GP37 from ALV-J and EAV-HP were also compared as described in Fig 3.

### Three tyrosine-based species of ALV-J Env

Because of the significance of the three motifs in cell signal transduction, we classified ALV-J Env into three species based on the distribution of YxxM, ITIM and ITAM-like in the CTD of GP37. The first species was named inhibitory Env and contains only ITIM without YxxM and ITAM-like. The second species was named bifunctional Env and contains both the ITIM and YxxM motifs. The third species has the YxxM and ITAM-like motifs and was named active Env. Based on this classification, 55, 92 and 2 of 149 of ALV-J Envs belong to the inhibitory Env, bifunctional Env and active Env, respectively. Additionally 17 of 18 EAV-HP only have ITIM without the YxxM and ITAM-like motifs and belong to the inhibitory Env. In the reported EAV-HP sequences from GenBank, only clone EAV-21-1 carried both the YxxM and ITIM motifs in GP37 and was a bifunctional Env. Similar to most of the EAV-HPs, the Env of HPRS-103, the ALV-J prototype strain, was also classified as inhibitory Env.

## Discussion

Although ALV-J GP37 is hypothesized to be less diverse than ALV-J GP85, our phylogenetic assay revealed that GP37 is clustered into five groups. In the classification, all EAV-HP GP37s were clustered closely in Group 5, indicating that EAV-HP GP37 is more conserved than ALV-J GP37. Unlike the other ALV-J GP37s, including the Chinese isolates, the GP37 of two ALV-J Chinese isolates, SD09DP04 and SX090912J, were clustered closely within the EAV-HP GP37 sequences, as described in Fig 2. The finding that the two Chinese isolates SD09DP04 and SX090912J carry the endemic GP85 and EAV-HP-like GP37 indicates that GP85 in ALV-J Env is prone to more variability than GP37. Because the GP37 of the two isolates identified in this study were closer to EAV-HP, the progenitor of ALV-J Env, than any other ALV-J isolates tested, further tracking of the GP37 evolution pattern of the two Chinese ALV-J isolates may reveal the genetic variant or host-adapted profile for ALV-J GP37. In addition, the molecular characteristics of the two isolates carrying GP85 endemic and EAV-HP-like GP37 also warrant further investigation.

Compared to the US isolates, the Chinese ALV-J isolates showed more variation in GP37, and some belonged to a unique group or subgroup. This finding is similar to the sequence analysis of ALV-J GP85, as previously described [11]. This is also consistent with the fact that ALV-J has experienced breakouts and extended its host range in China recently. Unlike the US isolates, the Chinese ALV-J isolates in GenBank were not only from meat-type chickens but also from egg-type chickens. This indicates that the host range or host pressure contributes to the genetic diversity of ALV-J GP37. Of the 15 sites in GP37 with viral host-adapted features identified in this study, 186K was unique for all EAV-HP GP37s. Furthermore, only ALV-J isolate SD09DP04 carries 186K in GP37, which was clustered closely within EAV-HP GP37. The 186R was dominant in ALV-J GP37. This indicates that 186K may be used as a potential biomarker for identifying EAV-HP from ALV-J. However, the roles of these sites and adapted mutations in ALV-J replication and pathogenesis must be further elucidated.

**Fig 4:**
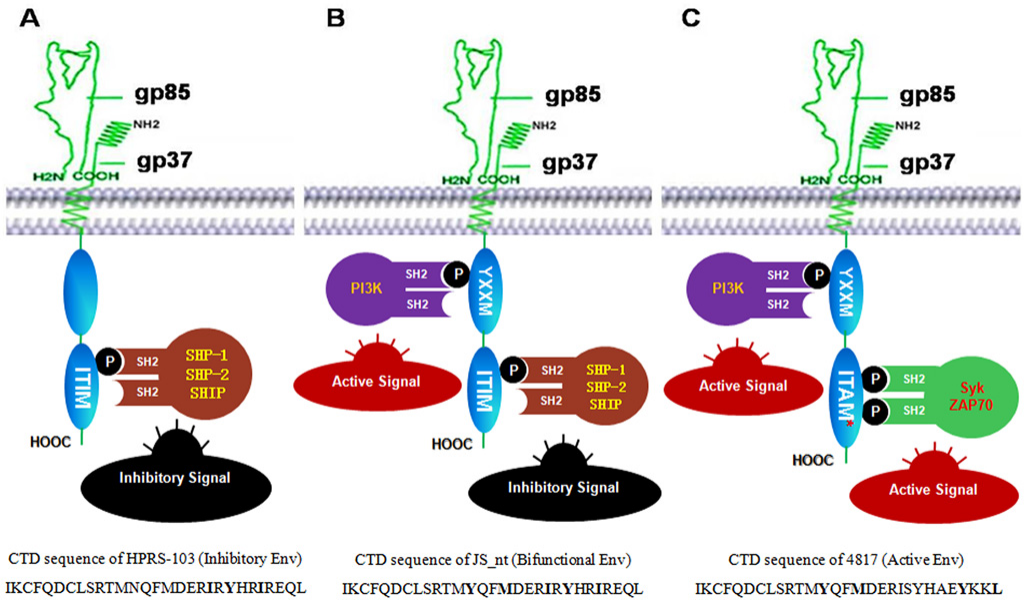
Models of signal transduction for the three types of ALV-J Env. A, The inhibitory Env with phosphorylated ITIM may recruit phosphatases, such as SHP1, SHP2 and SHIP, and then transduce an inhibitory signal. B, The bi-functional Env with phosphorylated ITIM may transmit an inhibitory signal as described in A, and the phosphorylated YxxM in bi-functional Env may activate the PI3K pathway to transduce an active signal. C, The active Env with the phosphorylated YxxM and ITAM-like motif may activate the PI3K pathway and Syk or ZAP70 pathway, respectively, to transmit an active signal. ITAM* indicated the ITAM-like motif.

Based on the findings and distribution of the three tyrosine-based motifs, YxxM, ITIM and ITAM-like, in the CTD of ALV-J GP37, ALV-J Env was first classified into three types. It should be noted that 17 of 18 EAV-HPs and ALV-J prototype isolate HPRS-103 Env belong to the inhibitory Env, whereas 92 of 149 ALV-J Envs belonged to the bifunctional Env. The inhibitory Env contains the NxxM sequence instead of YxxM. A single point mutation in NxxM could create the YxxM motif to convert inhibitory Env to bifunctional Env. This indicated that bifunctional Env may result from a point mutation of inhibitory Env under immune pressure during the replication of ALV-J, and the bifunctional Env showed a host adapted feature. Notably, the YxxM or ITIM or ITAM motif has also been reported in some other viral proteins, which contributes to viral pathogenesis and oncogenesis [24–28]. Therefore, we hypothesize that these motifs in ALV-J GP37 are functional and play vital roles in ALV-J pathogenesis and oncogenesis. And three models of signal transduction performed by the three types of Env were proposed as described in Fig 4. In Fig 4A, the inhibitory Env with phosphorylated ITIM may recruit phosphatases, such as SHP1, SHP2 and SHIP, and then dephosphorylate proteins to transmit an inhibitory signal. In bifunctional Env, ITIM may also conduct an inhibitory signal such as the inhibitory Env, whereas the phosphorylated YxxM may activate PI3K signaling to activate a kinase. For active Env, in addition to activating a signal transmitted by the phosphorylated YxxM, the phosphorylated ITAM-like motif may activate the Syk or ZAP70 kinase signaling pathway. As a normal component in chicken, EAV-HP may be a functional immune tyrosine inhibitory receptor or adaptor to control or maintain normal cell function. Furthermore, ITIM in ALV-J GP37 may play vital roles in immune-suppression induced by ALV-J infection, whereas the YxxM and ITAM-like motifs may contribute to ALV-J oncogenesis.

In conclusion, we report for the first time five groups of ALV-J GP37, 15 host-adapted mutations in ALV-J GP37, and the classification of ALV-J Env into three species based on the tyrosine-based motifs in GP37. These findings and the signal transduction models hypothesized for ALV-J Env provide novel insights not only for ALV-J and EAV-HP GP37 evolution, but also for elucidating the novel functions of ALV-J Env and EAV-HP, and their molecular mechanism.

## References

[1] Payne LN, Brown SR, Bumstead N, Howes K, Frazier JA, Thouless ME. A novel subgroup of exogenous avian leukosis virus in chickens. The Journal of general virology. 1991 4;72 (Pt 4):801–7. . 1849967 10.1099/0022-1317-72-4-801

[2] Payne LN, Howes K, Gillespie AM, Smith LM. Host range of Rous sarcoma virus pseudotype RSV(HPRS-103) in 12 avian species: support for a new avian retrovirus envelope subgroup, designated J. The Journal of general virology. 1992 11;73 (Pt 11):2995–7. . 1331300 10.1099/0022-1317-73-11-2995

[3] Payne LN, Nair V. The long view: 40 years of avian leukosis research. Avian pathology: journal of the WVPA. 2012;41(1):11–9. 10.1080/03079457.2011.646237 22845317

[4] Gao YL, Qin LT, Pan W, Wang YQ, Le Qi X, Gao HL, et al Avian leukosis virus subgroup J in layer chickens, China. Emerging infectious diseases. 2010 10;16(10):1637–8. Pubmed Central PMCID: 3294407. 10.3201/eid1610.100780 20875300 PMC3294407

[5] Chesters PM, Howes K, Petherbridge L, Evans S, Payne LN, Venugopal K. The viral envelope is a major determinant for the induction of lymphoid and myeloid tumours by avian leukosis virus subgroups A and J, respectively. The Journal of general virology. 2002 10;83(Pt 10):2553–61. . 12237439 10.1099/0022-1317-83-10-2553

[6] Brown DW, Robinson HL. Influence of env and long terminal repeat sequences on the tissue tropism of avian leukosis viruses. Journal of virology. 1988 12;62(12):4828–31. . Pubmed Central PMCID: 253609. 2846895 10.1128/jvi.62.12.4828-4831.1988 PMC253609

[7] Bai J, Payne LN, Skinner MA. HPRS-103 (exogenous avian leukosis virus, subgroup J) has an env gene related to those of endogenous elements EAV-0 and E51 and an E element found previously only in sarcoma viruses. Journal of virology. 1995 2;69(2):779–84. . Pubmed Central PMCID: 188642. 7815543 10.1128/jvi.69.2.779-784.1995 PMC188642

[8] Pan W, Gao Y, Qin L, Ni W, Liu Z, Yun B, et al Genetic diversity and phylogenetic analysis of glycoprotein GP85 of ALV-J isolates from Mainland China between 1999 and 2010: coexistence of two extremely different subgroups in layers. Veterinary microbiology. 2012 4 23;156(1–2):205–12. . 22101092 10.1016/j.vetmic.2011.10.019

[9] Jiang L, Zeng X, Hua Y, Gao Q, Fan Z, Chai H, et al Genetic diversity and phylogenetic analysis of glycoprotein gp85 of avian leukosis virus subgroup J wild-bird isolates from Northeast China. Archives of virology. 2014 7;159(7):1821–6. 10.1007/s00705-014-2004-8 24488027

[10] Wang Z, Cui Z. Evolution of gp85 gene of subgroup J avian leukosis virus under the selective pressure of antibodies. Science in China Series C, Life sciences / Chinese Academy of Sciences. 2006 6;49(3):227–34. . 16856491 10.1007/s11427-006-0227-y

[11] Cui Z, Du Y, Zhang Z, Silva RF. Comparison of Chinese field strains of avian leukosis subgroup J viruses with prototype strain HPRS-103 and United States strains. Avian Dis. 2003 Oct-Dec;47(4):1321–30. . 14708978 10.1637/6085

[12] Thu WL, Wang CH. Phylogenetic analysis of subgroup J avian leucosis virus from broiler and native chickens in Taiwan during 2000–2002. The Journal of veterinary medical science / the Japanese Society of Veterinary Science. 2003 3;65(3):325–8. . 12679561 10.1292/jvms.65.325

[13] Qin A, Lee LF, Fadly A, Hunt H, Cui Z. Development and characterization of monoclonal antibodies to subgroup J avian leukosis virus. Avian Dis. 2001 Oct-Dec;45(4):938–45. . 11785897

[14] Wu X, Qian K, Qin A, Shen H, Wang P, Jin W, et al Recombinant avian leukosis viruses of subgroup J isolated from field infected commercial layer chickens with hemangioma and myeloid leukosis possess an insertion in the E element. Veterinary research communications. 2010 10;34(7):619–32. Pubmed Central PMCID: 2931761. 10.1007/s11259-010-9436-8 20676760 PMC2931761

[15] Tamura K, Stecher G, Peterson D, Filipski A, Kumar S. MEGA6: Molecular Evolutionary Genetics Analysis Version 6.0. Molecular biology and evolution. 2013 12;30(12):2725–9. English. 10.1093/molbev/mst197 24132122 PMC3840312

[16] Crooks GE, Hon G, Chandonia JM, Brenner SE. WebLogo: a sequence logo generator. Genome research. 2004 6;14(6):1188–90. . Pubmed Central PMCID: 419797. 15173120 10.1101/gr.849004 PMC419797

[17] Yan Du ZC, Aijian Qin. Detection of avian leukosis virus subgroup J from commercial meat type chickens. China Poultry. 1999;3(1):1–4.

[18] Vanhaesebroeck B, Stephens L, Hawkins P. PI3K signalling: the path to discovery and understanding. Nature reviews Molecular cell biology. 2012 3;13(3):195–203. 10.1038/nrm3290 22358332

[19] Okkenhaug K, Vanhaesebroeck B. PI3K in lymphocyte development, differentiation and activation. Nature reviews Immunology. 2003 4;3(4):317–30. . 12669022 10.1038/nri1056

[20] Humphrey MB, Lanier LL, Nakamura MC. Role of ITAM-containing adapter proteins and their receptors in the immune system and bone. Immunological reviews. 2005 12;208:50–65. . 16313340 10.1111/j.0105-2896.2005.00325.x

[21] Ravetch JV, Lanier LL. Immune inhibitory receptors. Science. 2000 10 6;290(5489):84–9. . 11021804 10.1126/science.290.5489.84

[22] Billadeau DD, Leibson PJ. ITAMs versus ITIMs: striking a balance during cell regulation. The Journal of clinical investigation. 2002 1;109(2):161–8. . Pubmed Central PMCID: 150845. 11805126 10.1172/JCI14843 PMC150845

[23] Barrow AD, Trowsdale J. You say ITAM and I say ITIM, let's call the whole thing off: the ambiguity of immunoreceptor signalling. European journal of immunology. 2006 7;36(7):1646–53. . 16783855 10.1002/eji.200636195

[24] Willems L, Gatot JS, Mammerickx M, Portetelle D, Burny A, Kerkhofs P, et al The YXXL signalling motifs of the bovine leukemia virus transmembrane protein are required for in vivo infection and maintenance of high viral loads. Journal of virology. 1995 7;69(7):4137–41. . Pubmed Central PMCID: 189149. 7769672 10.1128/jvi.69.7.4137-4141.1995 PMC189149

[25] Wootton SK, Halbert CL, Miller AD. Sheep retrovirus structural protein induces lung tumours. Nature. 2005 4 14;434(7035):904–7. . Pubmed Central PMCID: 1401489. 15829964 10.1038/nature03492 PMC1401489

[26] Katz E, Lareef MH, Rassa JC, Grande SM, King LB, Russo J, et al MMTV Env encodes an ITAM responsible for transformation of mammary epithelial cells in three-dimensional culture. The Journal of experimental medicine. 2005 2 7;201(3):431–9. . Pubmed Central PMCID: 2213037. 15684322 10.1084/jem.20041471 PMC2213037

[27] Lanier LL. Viral immunoreceptor tyrosine-based activation motif (ITAM)-mediated signaling in cell transformation and cancer. Trends in cell biology. 2006 8;16(8):388–90. . 16815013 10.1016/j.tcb.2006.06.004

[28] Lagunoff M, Lukac DM, Ganem D. Immunoreceptor tyrosine-based activation motif-dependent signaling by Kaposi's sarcoma-associated herpesvirus K1 protein: effects on lytic viral replication. Journal of virology. 2001 7;75(13):5891–8. . Pubmed Central PMCID: 114304. 11390590 10.1128/JVI.75.13.5891-5898.2001 PMC114304

